# Gastrointestinal motility in microgravity: a critical review of multi-level mechanisms and model-dependent effects

**DOI:** 10.3389/fphys.2026.1930628

**Published:** 2026-08-20

**Authors:** Qian Niu, Tong Mu, Junhai Zhang, Yue Chen, Zhikun Guo, Yuanmin Zhu, Lin Li

**Affiliations:** 1 Department of Gastroenterology, Aerospace Center Hospital, Peking University Aerospace School of Clinical Medicine, Beijing, China; 2 The Second Clinical Medical School, Jiangxi Medical College, Nanchang University, Nanchang, Jiangxi, China

**Keywords:** constipation, gastric emptying, gastrointestinal motility, gut hormones, intestinal barrier, microgravity

## Abstract

**Background:**

Gastrointestinal motility disturbances rank among the most frequently reported medical complications of spaceflight. Astronauts experience delayed gastric emptying, erratic small intestinal transit and reduced colonic propulsion. The underlying mechanisms are multifactorial. Microgravity alters intra-abdominal physical mechanics, disrupts autonomic and enteric neural circuits, shifts gastrointestinal hormone secretion profiles, inflicts oxidative stress upon effector cells, and perturbs gut microbial communities. Cross-model comparisons reveal substantial disagreement, suggesting that no single ground-based *analog* fully captures the pathophysiology of orbital flight.

**Aim:**

To critically review how weightlessness affects gastric emptying, small intestinal transit and colonic motility; to critically evaluate contradictory findings across simulation platforms; and to delineate the neural, humoral, cellular and microbiological mechanisms involved.

**Methods:**

We searched PubMed, Web of Science and the NASA Technical Reports Server for articles published between January 1990 and June 2026 (last search 30 June 2026). Search terms included: “microgravity”, “weightlessness”, “spaceflight”, “gastrointestinal motility”, “gastric emptying”, “intestinal transit”, “gut microbiome”, “interstitial cells of Cajal” and “oxidative stress”. Studies using head-down bed rest, hindlimb unloading, clinorotation, parabolic flight and actual spaceflight were included. The review follows a critical narrative design; the full search strategy and the framework used to appraise the evidence are described in Section 1.1.

**Results:**

Altered-gravity studies suggest that gastrointestinal dysmotility may involve neurohumoral dysregulation, oxidative injury to interstitial cells of Cajal and smooth muscle, barrier dysfunction and altered enteric *signaling*; however, most mechanistic evidence derives from simulated models and has not been directly validated during human spaceflight. Direct human motility measurements remain sparse, and the evidence comprises a mixture of direct observations, model-dependent inferences and testable hypotheses. Cross-study agreement is poor: some head-down bed rest trials report accelerated small-bowel transit, whereas tail-suspension models and limited flight observations suggest motor suppression. These divergences may reflect model-specific confounding rather than a uniform effect of microgravity.

**Conclusion:**

Current ground-based models each capture only partial aspects of orbital GI pathophysiology. Future work should combine multi-omics profiling with next-generation simulation platforms to develop evidence-based countermeasures for long-duration missions.

## 1 Introduction

Human spaceflight is shifting from short-term low-Earth orbit missions to long-duration stays and deep-space exploration. This transition intensifies the challenge of maintaining crew health. The digestive system, central to energy homeostasis, undergoes marked functional changes in microgravity. Astronauts frequently report gastric discomfort, early satiety, abdominal bloating and constipation during flight, although the frequency and persistence of these symptoms are not consistently quantified across missions (Afonin et al., 2003). Beyond reducing quality of life, altered GI motility can impair the absorption of orally administered pharmaceuticals, a consideration of direct operational relevance.

Yet the evidence base remains fragmentary. Most data come from ground-based *analog*s rather than orbital observation. Head-down bed rest (HDBR) and hindlimb unloading (HLU) replicate cephalic fluid shifts and reduced mechanical loading. They do not, however, reproduce the free-floating state of abdominal viscera in three-dimensional space, vestibular sensory conflict or exposure to galactic cosmic radiation. These discrepancies generate genuinely contradictory findings. Certain HDBR studies describe faster small intestinal transit, yet tail-suspension rodent models and the limited human spaceflight data available point toward hypomotility (Prakash et al., 2015). The literature remains siloed across gastroenterology, aerospace medicine and physiology, impeding integrated mechanistic insight.

Clarification of terminology is warranted. In this review, “microgravity” denotes the near-weightless environment of orbital flight (~10^-6^ g). “Simulated microgravity” refers to ground-based models—principally HDBR, HLU and clinorotation—that recreate selected features of weightlessness. “Real spaceflight” encompasses data obtained during orbital, suborbital or interplanetary missions. Each platform introduces distinct physiological artifacts that must be weighed when interpreting results. This definitional rigor matters because conflation of model types has perpetuated confusion in the field.

**Figure 1:**
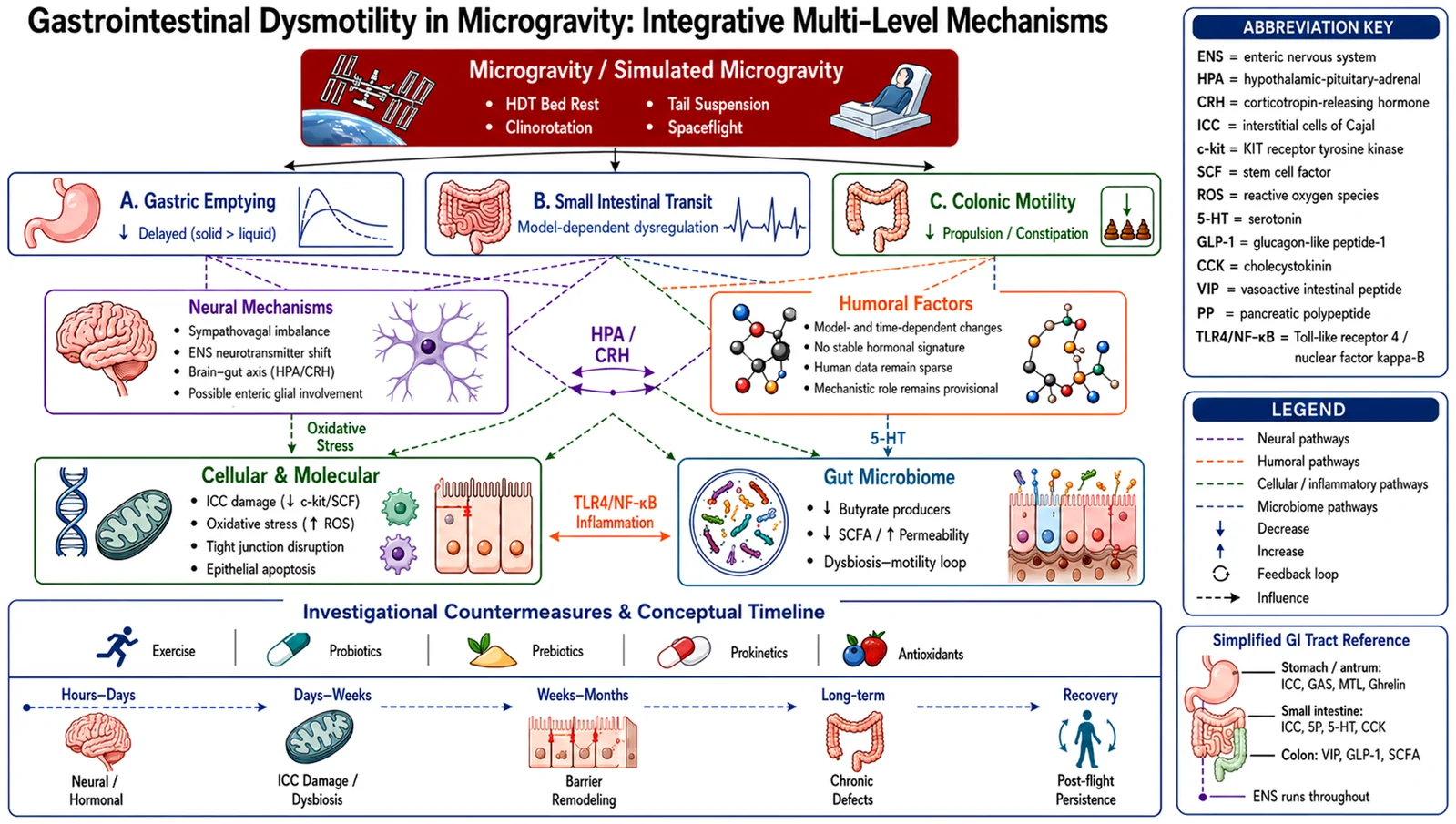
Gastrointestinal dysmotility in microgravity: integrative multi-level mechanisms. The schematic organizes proposed segmental effects—**(A)** delayed gastric emptying (more pronounced for solids than for liquids), **(B)** model-dependent dysregulation of small intestinal transit, and **(C)** reduced colonic propulsion—and links them to five mechanistic domains: autonomic and enteric neural dysregulation, gastrointestinal hormone imbalance, oxidative injury to interstitial cells of Cajal (ICC) and smooth muscle, epithelial barrier disruption, and gut microbial dysbiosis. Arrows indicate proposed directions of influence, and circular arrows indicate feedback loops (for example, the dysbiosis–dysmotility loop); the figure does not independently grade the certainty of each link—evidence strength and model limitations are described in the text and in **Table 1**. The lower band presents an approximate, hypothesis-generating temporal sequence rather than a validated timeline. Countermeasures shown are investigational and have not been validated against gastrointestinal motility endpoints during actual spaceflight. The artwork is original and was produced by the authors.

We have structured this review around three anatomical levels (gastric emptying, small intestinal transit, colonic motility) and four mechanistic dimensions (neural, humoral, cellular/molecular, microbial). Our analysis deliberately emphasizes model-specific artifacts and contradictory datasets that previous reviews have tended to gloss over (Sun et al., 2025). An integrative overview of the proposed multi-level mechanisms and investigational countermeasures is provided in **Figure 1**.

### 1.1 Review design and literature search

This article is a critical narrative review. It is not a systematic or scoping review, and no protocol was registered. Its purpose is to integrate mechanistic evidence across experimental platforms and to confront contradictory findings, rather than to enumerate all eligible studies exhaustively. We searched PubMed, Web of Science and the NASA Technical Reports Server for articles published between January 1990 and June 2026; the last search was performed on 30 June 2026. The core string was adapted to each database: (“microgravity” OR “weightlessness” OR “spaceflight” OR “head-down bed rest” OR “hindlimb unloading” OR “clinorotation” OR “parabolic flight” OR “hypergravity”) AND (“gastrointestinal motility” OR “gastric emptying” OR “intestinal transit” OR “colonic motility” OR “constipation” OR “gut microbiome” OR “interstitial cells of Cajal” OR “intestinal barrier” OR “gut hormone” OR “oxidative stress”). Reference lists of retrieved articles were screened for additional records. No formal language restriction was applied; English- and Chinese-language reports were included when the full text could be adequately assessed. Conference proceedings and one doctoral dissertation were retained when they contained primary data unavailable elsewhere, and this is indicated in **Table 1**. Two authors screened titles and abstracts independently, and disagreements were resolved by discussion with a third author.

**Table 1:** Summary of the principal studies reviewed, organized by evidence tier and gastrointestinal domain.

Study [Ref.]	Subjects/platform	Duration	GI outcome(s) assessed	Principal findings	Major limitations
Human studies — spaceflight and ground-based *analog*s					
Prakash et al., 2015	Human; 6° head-down-tilt bed rest	5 days	Gastric emptying; oro-cecal transit (lactulose H_2_ breath test)	Gastric emptying unchanged; small-bowel and colonic transit accelerated by day 2; partial normalization by day 5	Small sample; short exposure; fluid-shift confounding
Afonin et al., 2003	Human; Mir orbital flight (12 cosmonauts)	Months	Gastric emptying (glucose–lactose load); pancreatic and gallbladder response	Gastric emptying delayed throughout mission; diminished pancreatic and gallbladder responsiveness	No in-flight control group; surrogate endpoints; small n
Afonin et al., 2011	Human; dry immersion	5 days	Liquid gastric emptying; segmental chyme transit	Liquid emptying unchanged; small-bowel transit accelerated; colonic transit slowed	Short exposure; small n
Harm et al., 2002	Human; Space Shuttle	Acute flight phase	Gastric myoelectric activity (EGG)	Reduced postprandial slow-wave power; dysrhythmias during nausea episodes	Very small n; motion-sickness confounding; EGG is not a direct measure of emptying
Holwerda et al., 2016	Human; −20° head-down tilt	Acute (hours)	Gastric emptying; postprandial plasma amino acids	Slowed gastric emptying; delayed amino-acid absorption	Acute postural study, not a validated microgravity *analog*; liquid meal only
Riepl et al., 2002	Human; EUROMIR-94 mission	Acute + 4 weeks	Plasma gastrointestinal hormones	Acute: VIP, PP, motilin, secretin ↑, CCK ↓; chronic: CCK, motilin, VIP, neurotensin, insulin ↑	Single mission; small n; plasma levels only
Voorhies et al., 2019	Human; ISS astronauts (n = 9; in-flight stool n = 5)	Long-duration	Faecal microbiome; circulating cytokines	Alpha diversity/richness increased in 4 of 5 stool-sampled astronauts; community composition shifted; Akkermansia and Ruminococcus declined; changes largely reversible after return	Small sample; *fecal* sampling only; shared diet and environment
La Barbera et al., 2026	Human; ISS (n = 52)	Long-duration	Longitudinal plasma metabolomics	~40 circulating metabolites altered; increased microbial protein fermentation, consistent with prolonged intestinal transit	Associational; transit not directly measured
Computational studies					
Li et al., 2024	CFD simulation of the human stomach	—	Chyme mixing and emptying under reduced gravity	Mixing efficiency impaired; homogeneous foam-like chyme increases resistance to antral grinding	In silico; no tissue-level biology
Animal models — gastric and small-intestinal outcomes					
Li et al., 2013	Rat; tail suspension	Days–weeks	Antral c-kit protein/mRNA; serum gastrin and motilin	ICC marker expression ↓; gastrin early surge then decline; motilin progressive ↑	Restraint-stress confound; rodent
Li et al., 2026	Rat; tail suspension	21 days	Gastric emptying; intestinal propulsion; brain–gut peptides	Emptying and propulsion inhibited; SP/GAS/MTL ↓; VIP/CCK/CGRP/nesfatin-1 ↑	Simulated model; rodent
Chen et al., 2012	Rat; tail suspension	14–21 days	Small-intestinal propulsion; gastric residue; ghrelin, VIP	Propulsion weakened; residue increased; ghrelin ↓ with partial recovery by day 21	Simulated model; rodent
Peana et al., 2008	Rat; random positioning machine	—	Intestinal transit; iNOS/COX-2 expression	Transit slowed; iNOS ↑, COX-2 ↓	Conference proceedings; limited methodological detail
Lu et al., 2002	Rat; tail suspension	—	P-glycoprotein and CYP450 expression	P-gp expression altered	Indirect relevance to motility
Chen et al., 2008b	Rat; tail suspension	14–21 days	Small-intestinal mucosal morphology (light and electron microscopy)	Villus number and height ↓; microvilli shortened; glycocalyx thinner	Descriptive; rodent
Chen et al., 2011	Rat; tail suspension	14–21 days	Occludin and ZO-1 (immunohistochemistry, RT-qPCR)	Progressive tight-junction protein loss with patchy distribution by day 21	Rodent
Zhu et al., 2004	Rat; tail suspension	14–28 days	Antral somatostatin and IL-2 immunoreactivity	Both significantly decreased	Rodent; regional sampling
Wang et al., 2007	Rat; tail suspension	28 days	Gastric mucosal leptin and leptin receptor	Both increased	Rodent
Animal models — colonic, barrier, vascular and immune outcomes					
Jin et al., 2018	Rat; tail suspension	—	Mucosal barrier (diamine oxidase, D-lactate, tight junctions)	Barrier dysfunction; permeability ↑	Simulated model; barrier rather than motility endpoint
Li, 2008	Rat; tail suspension	0.5–21 days	Mucosal apoptosis; NF-κB; immune indices	Early epithelial and lymphocyte apoptosis; sIgA ↓; partial recovery by day 7	Doctoral dissertation; single center
Li et al., 2008	Rat; tail suspension	0.5–21 days	NF-κB expression in intestinal mucosa	NF-κB ↑ from 0.5 day, peak at day 2, sustained to day 21	Rodent
Li and Ge, 2014	Rat; tail suspension ± DSS colitis	2 weeks	Colonic innate immunity	Neutrophil activity and IL-1 ↑; aggravated DSS colitis	Conference abstract; not fully peer-reviewed
Zhang et al., 1998	Rat; tail suspension	14 days	Mesenteric arteriolar reactivity	Contractile responses to noradrenaline and KCl ↓	Vascular endpoint; rodent
Dunbar et al., 2000	Rat; hindlimb unweighting	—	Mesenteric venous capacitance and reactivity	Reduced vasoconstrictor reactivity	Vascular endpoint
Shi et al., 2017	Mouse; hindlimb unloading	≥3 days	Colonic epithelium; gut microbiota	Goblet-cell and epithelial-homeostasis changes; microbial compositional shifts within days	Rodent; motility not directly measured
Benvenuti et al., 2026	Differentiated intestinal epithelial monolayers; simulated Martian gravity (0.38 g)	—	Barrier integrity; tight-junction components	Barrier function and selected tight-junction components altered	*In vitro* partial-gravity model; no motility endpoint
Microbiome and intervention studies					
Casero et al., 2017	Mouse; space-type radiation	—	Gut microbiome and metabolome	Multimodal microbiome and metabolome shifts	Radiation only; no unloading
Suzuki et al., 2024	Mouse (ApcMin/+); radiation + simulated microgravity	—	Intestinal tumorigenesis	Joint tumour-promoting effect of the two stressors	Cancer endpoint, not motility or microbiome endpoint
Wang et al., 2024	Rat; simulated weightlessness	—	Gut microbiota; bile-acid metabolites	Altered bile-acid metabolite spectrum; glycine-conjugated bile acids ↓	Simulated model
Alauzet et al., 2019	Mouse; 2 g/3 g centrifugation	21 days	Caecal microbiota; mucosal integrity	3 g altered diversity and community structure; mucosa intact	Hypergravity, not microgravity; cecal sampling
Thompson et al., 2025	Mouse (female); 3 g ± GOS/polydextrose diet	4 weeks	Gut microbiome; neutrophil-to-lymphocyte ratio	Prebiotic diet prevented hypergravity-induced dysbiosis; NLR reduced	Hypergravity model; females only
Zhang et al., 2025	Rat; simulated microgravity + Bifidobacterium lactis BLa80	28 days	Faecal and mucosal flora; SCF/c-Kit *signaling*	Microbiota, mucosal status and SCF/c-Kit-related *signaling* improved	Preclinical; motility and ICC pacemaker function not directly measured
Zang et al., 2025	Rat; tail suspension + Hericium erinaceus polysaccharides	—	Gastrointestinal function; mucosal barrier	Dysfunction ameliorated	Preclinical intervention
Chen et al., 2026	Rat; long-term tail suspension	Long-term	Gut–liver axis; hepatic metabolism	Hepatic metabolic-enzyme and lipid-metabolism disturbance	Simulated model
Xiong et al., 2025	Mouse; three-dimensional clinostat	42 days	Microbiota; liver and brain metabolic profiles	Dysbiosis associated with cross-organ metabolic changes	Simulated model; causal direction and human relevance unresolved
*In vitro* studies					
Alvarez et al., 2019	Human intestinal epithelial cells; clinostat	9–11 days	Barrier function (TEER); ZO-1/occludin	Tight-junction proteins ↓ at day 9, recovered by day 11; sustained functional barrier defect	*In vitro*; no systemic neurohumoral inputs

GE, gastric emptying; ICC, interstitial cells of Cajal; HDT, head-down tilt; ISS, International Space Station; NLR, neutrophil-to-lymphocyte ratio; TEER, transepithelial electrical resistance.

The evidence base spans human orbital observations, human ground-based *analog*s, animal models, cell culture and computational studies. Formal risk-of-bias tools developed for systematic reviews of homogeneous designs are therefore not directly applicable. Instead, we appraised each source qualitatively along four dimensions: internal validity (design, sample size, objectivity of endpoints), ecological validity (fidelity to the spaceflight environment), model-specific bias (for example, restraint stress in hindlimb unloading and fluid-shift confounding in head-down tilt), and translational relevance to human spaceflight. This appraisal informed the limitations column of **Table 1** and the weight given to each finding in the text. Throughout the review we distinguish, as far as the underlying data allow, between direct observations, mechanistic inferences drawn from *analog* models, and hypothesis-generating speculation. .

## 2 Effects of weightlessness on gastric emptying

### 2.1 Delayed gastric emptying: observations and conflicting evidence

Gastric emptying—the process by which gastric contents enter the duodenum—is governed by coordinated antral peristalsis, pyloric sphincter tone, and duodenal feedback. Multiple studies have reported that weightlessness delays this process, although the effect size varies with meal composition and flight duration. Early Space Shuttle astronauts experienced marked gastric emptying delays during the initial flight phase, with both liquid and solid meals exhibiting prolonged transit times compared to ground-based measurements. Parabolic flight experiments similarly demonstrated reduced emptying rates during brief weightlessness episodes, particularly after high-fat meals.

Long-duration spaceflight data support the persistence of this phenomenon. A comprehensive monitoring study of twelve cosmonauts aboard the Mir space station, using a glucose-lactose load test, revealed delayed gastric emptying throughout the mission alongside diminished pancreatic and gallbladder responsiveness (Afonin et al., 2003). Because this study lacked an in-flight control group and relied on indirect physiological endpoints, these observations suggest—but do not establish—that emptying delay is a sustained disturbance rather than a transient adaptive response.

Nevertheless, model-specific discrepancies demand careful scrutiny. Afonin and colleagues, using dry immersion to simulate microgravity, found no significant alteration in liquid gastric emptying, whereas chyme transit accelerated through the small intestine yet slowed through the colon (Afonin et al., 2003; Afonin et al., 2011). Prakash et al. reported comparable findings with 6° head-down tilt (HDT) bed rest: unchanged gastric emptying but accelerated small bowel and colonic transit, with maximal changes evident by day two of HDT and partial normalization by day five (Prakash et al., 2015). These seemingly paradoxical results do not negate the influence of weightlessness on gastric emptying. Rather, they reflect methodological differences. Liquid emptying is driven primarily by gastric fundus tone and pyloric pressure gradients, mechanisms that depend little on gravitational assistance. Solid emptying, by contrast, depends critically on antral grinding and gravity-dependent chyme stratification, rendering it more vulnerable to weightlessness. Moreover, although the HDT model reproduces cephalic fluid shifts, it cannot replicate the three-dimensional flotation of abdominal organs characteristic of true weightlessness. This limitation may cause HDT studies to underestimate the inhibitory effects on solid gastric emptying.

Recent computational fluid dynamics (CFD) modeling offers a mechanistic reconciliation. A 2024 simulation of human gastric emptying under reduced gravity demonstrated that lower gravitational levels impair mixing efficiency between chyme and digestive secretions. Density stratification disappears; instead, a homogeneous foam-like mixture forms, substantially increasing resistance to antral grinding and pyloric coordination (Li et al., 2024). This biomechanical explanation bridges the apparent contradiction between preserved liquid emptying and delayed solid emptying: the effect of weightlessness on gastric emptying is contingent upon the physicochemical properties of the meal. It remains, however, a biomechanical hypothesis: the cited studies did not standardize meal properties sufficiently to establish a gravity-specific liquid–solid contrast, and in silico evidence reconciles the observations conceptually rather than demonstrating a clinical mechanism.

The dependence on meal composition highlights a fundamental limitation in the field. Early studies rarely standardized meal viscosity, nutrient density, or particle size across protocols, making cross-study comparisons problematic. The development of standardized meal paradigms—validated in both ground-based simulators and orbital platforms—remains an urgent methodological priority.

### 2.2 Mechanisms of gastric emptying delay: from biomechanics to cellular pacemaking

Weightlessness-induced gastric emptying delay arises from the interplay of several hierarchical mechanisms. Cephalad fluid transfer engenders visceral venous congestion and passive gastric wall distension. Mechanoreceptor activation triggers reflexive inhibition of antral contractions and increased pyloric tone. Simultaneously, the stomach loses its gravitational anchor. The gastric fundus rises with the diaphragm, disrupting the normal pressure gradient between gastric and duodenal lumens and compromising pyloric coordination.

Electrophysiological evidence supports these mechanical explanations. Harm et al. recorded electrogastrography (EGG) signals from astronauts during flight and observed reduced postprandial slow-wave power during the initial flight phase. During nausea episodes, gastric dysrhythmias increased, with elevated ratios of abnormal slow-wave to tachygastric activity (Harm et al., 2002). Because electrogastrography is a body-surface measure rather than a direct measure of emptying, these findings demonstrate altered gastric electrical activity and possible pacemaker suppression, but cannot by themselves establish delayed emptying. Ground-based simulation studies complement this observation: Holwerda et al. confirmed that −20° head-down tilt significantly slows gastric emptying and delays postprandial amino acid absorption (Holwerda et al., 2016). The −20° protocol, however, was an acute body-position experiment rather than a validated microgravity *analog*, and differed from the 6° HDBR studies in exposure duration, meal and endpoint; the contrast between the two should therefore not be interpreted as a tilt-angle dose-response relationship.

At the cellular level, damage to interstitial cells of Cajal (ICC) represents a key mechanism underlying gastric dysmotility. Li and colleagues, using a tail-suspension rat model, demonstrated significant reductions in c-kit protein and mRNA expression within the gastric antrum muscular layer—molecular markers specific to ICC pacemaker function (Li et al., 2013). This finding reframes gastric emptying delay from a purely functional disturbance to one with structural cellular underpinnings. The tail-suspension model, however, applies sustained mechanical stress to the abdominal wall that true weightlessness does not. Whether ICC damage occurs via analogous mechanisms in orbit—where hydrostatic unloading rather than mechanical compression predominates—remains unresolved. Human validation should instead prioritize non-invasive approaches—standardized emptying tests, body-surface slow-wave mapping and longitudinal biomarkers.

### 2.3 Hypergravity: the overlooked opposite pole of gravitational loading

Spaceflight exposes the gastrointestinal tract not only to weightlessness but also to hypergravity during launch, re-entry and the pull-out phases of parabolic flight. Altered gravity is therefore a continuous exposure rather than a binary one, and we surveyed the hypergravity literature for the three themes of this review. Direct evidence is strikingly scarce: we identified no original study examining gastric emptying rate, small intestinal transit time, colonic motility or enteric nervous system function under hypergravity. The nearest evidence concerns gastric secretion and mucosal integrity. In rats, acute exposure to +10 Gz reduced gastric juice volume and total acidity without changing plasma gastrin, pointing to a sympathetically driven mechanism independent of gastrin (Yoon and Kim, 2017); the effect was abolished by adrenalectomy, implicating adrenal catecholamines as the principal mediator (Na and Kim, 2017). Positive acceleration also aggravated ethanol-induced gastric mucosal injury in a G-load-dependent manner, with increased mucosal malondialdehyde and reduced superoxide dismutase activity, suggesting oxidative stress involvement (Li et al., 2015). At the microbial level, 21 days of centrifugation at 3 g, but not 2 g, altered cecal microbial diversity and community structure in mice without compromising mucosal integrity (Alauzet et al., 2019). Whether these secretory, mucosal and microbial effects translate into altered propulsive motility is unknown. Because launch and re-entry hypergravity bracket every period of orbital weightlessness, motility profiling across the full gravity spectrum—ideally within the same subjects or animals—represents a concrete, currently empty niche for future investigation (see Section 9.2).

## 3 Effects of weightlessness on small intestinal propulsion

### 3.1 Small intestinal transit: acceleration or impairment?

Small intestinal propulsion is essential for nutrient digestion and absorption. Research findings regarding weightless effects on small bowel transit remain notably discordant—a divergence that itself exposes the limitations of current models and detection methodologies.

Some head-down tilt studies have reported shortened small intestinal transit times. Prakash et al., employing 6° HDT, observed earlier peak hydrogen exhalation in lactulose breath tests by day two of bed rest, suggesting accelerated transit. Stool consistency softened and daily defecation frequency increased, indicating accelerated colonic transit as well (Prakash et al., 2015). These changes likely reflect the altered intestinal hydrostatic pressure and reflexive peristaltic enhancement caused by cephalad fluid redistribution in the HDT position rather than a true microgravity state. In actual weightlessness, the intestine lacks gravitational anchoring. Intestinal wall tension signals become distorted and local reflex coordination is disrupted, producing kinetic characteristics fundamentally distinct from HDT.

Conversely, a larger body of evidence suggests inhibitory or disordered small intestinal motility under weightless or simulated weightless conditions. The tail-suspension model—a classic approach for simulating hindlimb unloading—frequently yields results diverging from HDT studies. Li et al. demonstrated that after 21 days of tail suspension, rats exhibited inhibited gastric emptying and intestinal propulsion alongside brain-gut peptide imbalance: reduced excitatory neurotransmitters including substance P (SP), gastrin (GAS), and motilin (MTL), alongside elevated inhibitory mediators such as vasoactive intestinal peptide (VIP), cholecystokinin (CCK), calcitonin gene-related peptide (CGRP), and nesfatin-1 (Li et al., 2026). This study broadens the spectrum of weightlessness-related enteric neuropeptide dysregulation, implicating novel peptides such as nesfatin-1 in the pathophysiology of spaceflight-induced gastrointestinal dysmotility. Chen and colleagues, using the same model, confirmed weakened small intestinal propulsion and increased gastric residue after 14 and 21 days of simulated weightlessness (Chen et al., 2012).

Taken together, the literature does not support a single directional effect on small-intestinal transit; the most defensible reading is model-dependent dysregulation. Where a directional tendency is discernible, the indirect evidence leans towards dysrhythmic hypomotility during actual weightlessness, but this inference rests on cross-model comparison rather than direct within-study contrast. The transit acceleration observed in HDT models probably represents an acute compensatory response to positional fluid shifts that attenuates or reverses with prolonged exposure. Tail-suspension data and actual flight observations align more closely with the hypokinetic features of chronic weightlessness. This interpretive framework remains provisional: no study has directly compared HDT, tail-suspension, and orbital outcomes within a unified experimental design. Such cross-model standardization represents an essential next step.

### 3.2 Molecular regulatory mechanisms of small intestinal propulsion

Small intestinal transit is regulated by numerous mediators with contractile or relaxant activity. At the molecular level, simulated weightlessness in rats significantly elevates inducible nitric oxide synthase (iNOS) content while reducing cyclooxygenase-2 (COX-2) expression in small intestinal tissue; COX-1 levels remain unchanged (Peana et al., 2008). iNOS-derived nitric oxide participates in pathological transit regulation, whereas COX-2-derived prostaglandins function as contractile agents promoting gastrointestinal motility. Simulated weightlessness therefore appears to inhibit prostaglandin-mediated contraction by stimulating iNOS and suppressing COX-2, thereby slowing small intestinal movement. This finding advances the mechanistic understanding from macroscopic physiology to specific enzymatic pathways, though its generalizability to humans remains unverified.

P-glycoprotein (P-gp) expression at the small intestinal epithelium also changes under weightless conditions (Lu et al., 2002). While P-gp primarily mediates drug efflux, its functional alteration reflects broader epithelial disturbance in the weightless environment that may contribute to discoordinated propulsion.

The net effect on small intestinal propulsion emerges from the convergence of biomechanical alteration, neural dysregulation, and molecular pathway derangement. The migrating motor complex (MMC)—the interdigestive “housekeeping” rhythm—suffers particular disruption. Phase III contractions clear residual content and bacteria from the small bowel (Deloose et al., 2012); whether their cyclical pattern is suppressed during weightlessness has not been measured directly, and the consequent risk of small intestinal bacterial overgrowth (SIBO) remains a testable hypothesis.

The predominance of rat-based molecular studies, however, imposes significant translational constraints. Species differences in enteric neurotransmitter profiles, MMC cycling frequency, and microbial ecology limit direct extrapolation to human spaceflight. Rodent studies have been indispensable for mechanistic hypothesis generation, but validation in human subjects using minimally invasive motility assessment tools—wireless capsule motility, serum biomarker panels, or post-flight tissue sampling—remains the critical next phase.

## 4 Effects of weightlessness on colonic motility

### 4.1 Colonic propulsion impairment and constipation: core symptoms

Colonic motor dysfunction ranks among the most frequently reported gastrointestinal complaints in spaceflight. Constipation occurs commonly during orbital missions, with symptoms most pronounced during the initial adaptation phase and throughout long-duration flights (Afonin et al., 2003). Astronauts report reduced bowel frequency, hard stool consistency, and difficult evacuation. Ground-based analog studies corroborate these observations: hindlimb-unloaded rodents show prolonged colonic transit and reduced fecal water content (Chen et al., 2008b; Jin et al., 2018).

The etiology of spaceflight constipation is multifactorial. Fluid shifts towards the head alter splanchnic hemodynamics. Inadequate fluid intake desiccates intraluminal contents. Dietary fiber consumption typically falls below recommended levels. Dysbiosis alters the colonic metabolic milieu. Practical constraints of space toilet facilities further suppress the defecation reflex (Afonin et al., 2003). These operational factors notwithstanding, biological mechanisms remain central. Direct manometric evidence from altered-gravity models is lacking; claims of diminished smooth-muscle contractile force, reduced high-amplitude propagating contractions or an attenuated recto-anal inhibitory reflex should therefore be regarded as extrapolations awaiting direct measurement (Jin et al., 2018). The relative contribution of each factor remains poorly quantified; existing studies rarely control simultaneously for dietary intake, fluid balance, and gravitational loading, making it difficult to isolate primary from secondary causes.

Low-grade colonic inflammation represents an important but frequently overlooked contributor to weightlessness-induced dysmotility. Li et al. reported that two weeks of hindlimb unloading in rats increased neutrophil count and activity in colonic tissue, upregulated IL-1 expression, and enhanced pro-inflammatory cytokine secretion from peritoneal macrophages (Li and Ge, 2014). When combined with dextran sulfate sodium-induced colitis, tail-suspended animals exhibited more severe hematochezia and heavier inflammatory infiltrates than ground controls (Li and Ge, 2014). Weightlessness thus appears to activate low-grade inflammation rather than merely depressing motility. This inflammatory state alters smooth muscle excitability and sensitivity—an inflammation-motility coupling mechanism that may explain why astronaut constipation often proves refractory to simple dietary fiber supplementation.

### 4.2 Alterations in colonic absorption and secretion

The colon maintains water and electrolyte absorption, stool desiccation, and propulsive peristalsis. Microgravity disrupts these functions at several levels. Epithelial barrier integrity deteriorates, with reduced tight-junction protein expression and rising circulating diamine oxidase and D-lactate—markers of enhanced paracellular passage (detailed in Section 7.2) (Jin et al., 2018). The clinical significance of these changes in the colon proper, as opposed to the small intestine, remains under active investigation.

Mucus layer alterations further compromise colonic homeostasis. Goblet cell-derived MUC2 constitutes the principal structural component of the protective mucus barrier. In simulated microgravity, goblet cell numbers decline, MUC2 expression drops, and mucus layer thickness decreases, permitting increased bacterial translocation (Chen et al., 2008b). Vasoactive intestinal peptide (VIP) expression rises under similar conditions (Furness, 2012). VIP stimulates intestinal water and electrolyte secretion while concurrently inhibiting smooth muscle contraction, further disturbing the absorption-secretion balance and propulsive function. Whether VIP upregulation represents a primary pathogenic event or a compensatory response to altered luminal conditions has yet to be established.

## 5 Neural mechanisms underlying weightlessness-induced gastrointestinal dysmotility

### 5.1 Autonomic dysfunction: sympathovagal imbalance

Gastrointestinal motility depends upon precise autonomic coordination. Autonomic adaptation during weightlessness is complex and should not be reduced to a uniform pattern of sympathetic augmentation with vagal withdrawal. Human studies report time- and context-dependent changes: Iwase et al. attributed the reduction in heart rate during microgravity to sympathetic withdrawal rather than vagal withdrawal (Iwase et al., 1999), while altered vagal baroreflex control has also been documented in space (Eckberg et al., 2010). These cardiovascular findings make an autonomic contribution to gastrointestinal symptoms plausible, but they do not directly establish regional motility effects.

Space motion sickness compounds this autonomic disturbance through abnormal vestibulo-autonomic reflexes. Otolithic organs lose gravitational loading in orbit, generating sensory conflict with visual and proprioceptive inputs. Central projections from the vestibular nuclei to the dorsal vagal nucleus produce irregular fluctuations in vagal tone (Yates and Bolton, 2014; Yates et al., 2016). This sympathovagal imbalance constitutes a key neurogenic substrate for the early-phase delay in gastric emptying and the broader gastrointestinal dysmotility observed in the initial days of spaceflight.

The autonomic framework alone cannot account for the persistence of constipation and gastroparesis-like symptoms during chronic spaceflight. Sympathovagal indices show partial normalization after approximately one to two weeks in orbit, yet colonic dysfunction continues for months. This temporal mismatch indicates that humoral factors, cellular adaptation, and microecological changes assume progressively greater importance during extended missions—a shift in pathophysiological emphasis that current countermeasures largely fail to address.

### 5.2 Enteric nervous system dysfunction, enteric glial cells, and visceral hemodynamic alterations

The enteric nervous system (ENS)—comprising the myenteric (Auerbach’s) and submucosal (Meissner’s) plexuses—maintains capacity for independent local reflex regulation (Spencer and Hu, 2020). In weightlessness, the mechanical and chemical input signals received by the ENS change fundamentally. Organ flotation and abnormal bowel wall distension reduce release of excitatory neurotransmitters (acetylcholine, substance P) while enhancing release of inhibitory mediators (nitric oxide, VIP) (Furness, 2012; Spencer and Hu, 2020). This neurotransmitter shift pushes the ENS towards a hypomotile state, directly suppressing smooth muscle contraction.

Enteric glial cells (EGCs) have emerged as critical regulators of ENS function. These cells provide structural support to enteric neurons, modulate synaptic neurotransmission through calcium-dependent gliotransmitter release, and participate in innate immune responses within the gut wall. EGCs express functional receptors for neurotransmitters, cytokines, and microbial products, enabling bidirectional communication with both neurons and the luminal environment. In inflammatory states, activated EGCs release S100B protein and pro-inflammatory cytokines that alter neuronal excitability. EGC involvement in spaceflight dysmotility is biologically plausible, particularly where inflammation and barrier dysfunction coexist. However, we identified no study that directly measured enteric-glial activation in a validated altered-gravity gastrointestinal model; EGC-directed treatment should therefore be regarded strictly as a future research concept.

The ENS forms a functional syncytium with interstitial cells of Cajal (ICC) via gap junctions. Aberrant ENS output disrupts ICC pacemaker function; conversely, ICC rhythm disturbances impair ENS coordination efficiency. This local neuro-pacemaker feedback loop progressively degrades propulsive organization (Dunbar et al., 2000). The decline of ICC integrity under inflammation and oxidative stress is detailed in Section 7.1.

Visceral hemodynamic alterations constitute an underappreciated pathophysiological substrate for gastrointestinal dysmotility. Zhang et al. demonstrated that simulated microgravity significantly diminished contractile responses of rat mesenteric arterioles to noradrenaline and potassium chloride (Zhang et al., 1998). Dunbar et al. reported reduced vasoconstrictor reactivity of mesenteric vessels to sympathetic stimulation (Dunbar et al., 2000). This vascular hyporesponsiveness leads to relative mucosal hypoperfusion, impaired smooth muscle energy metabolism, and weakened contractile capacity. ICC pacemaker activity is particularly oxygen-dependent; even modest reductions in tissue perfusion produce disproportionate slowing of slow-wave frequency. The combination of autonomic dysregulation, ENS-ICC uncoupling, glial activation, and visceral hypoperfusion creates converging pathways through which weightlessness compromises gastrointestinal propulsion.

### 5.3 Brain-gut axis disruption

The confined, isolated environment of spacecraft, coupled with circadian disruption and mission-related psychological stress, exerts profound effects on gastrointestinal function through the brain-gut axis. Stress activates the hypothalamic-pituitary-adrenal (HPA) axis in a characteristic biphasic pattern: an initial rapid surge of corticotropin-releasing hormone (CRH) from the paraventricular nucleus within minutes, followed by sustained elevation lasting hours to days (Taché and Bonaz, 2007). CRH acts directly on myenteric neurons to inhibit gastric emptying and increases colonic permeability through mast cell-dependent mechanisms (Taché and Bonaz, 2007). Terrestrial studies show that CRH pathways can alter gastric and colonic motor and barrier function (Taché and Bonaz, 2007; Stengel and Taché, 2009); direct in-flight measurements of intestinal CRH *signaling* and associated motor endpoints, however, are scarce. This prolonged CRH exposure differs qualitatively from acute stress responses. Chronic stimulation downregulates CRH receptor expression in the colon while upregulating central receptor populations, creating a central-peripheral dissociation that may underlie the persistence of gastrointestinal symptoms despite apparent physiological adaptation.

Vagal afferent signaling undergoes substantial remodeling in space. Mechanoreceptors in the gastric wall, normally tuned to detect food-induced distension, fire less frequently when gastric contents float freely in microgravity. Chemoreceptors sensitive to luminal nutrient composition send aberrant signals because altered gastric emptying changes the temporal pattern of nutrient delivery to the proximal small intestine. Polymodal receptors that respond to chemical, mechanical, and thermal stimuli show altered thresholds under conditions of autonomic imbalance (Steinert et al., 2017). These distorted afferent signals reach the nucleus tractus solitarius, where central integration generates inaccurate perceptions of satiety and hunger. Astronauts frequently report early satiety and reduced appetite. These phenomena diminish meal volume and frequency, thereby depriving the gut of the mechanical stimulation necessary for normal enteroceptive reflexes. This sensory misrepresentation raises an important question: to what extent are spaceflight gastrointestinal symptoms driven by peripheral physiological changes versus central misinterpretation of afferent signals?

Psychological stress further perturbs the gut microbiota-brain communication axis. Chronic HPA activation alters the relative abundance of Firmicutes and Bacteroidetes, reduces short-chain fatty acid (SCFA) production, and increases intestinal permeability (Stengel and Taché, 2009; Steinert et al., 2017). Reduced short-chain fatty acid availability then compromises epithelial barrier integrity and permits bacterial component translocation (detailed in Section 8.3). The resulting microbial and pathogen-associated signals reach central stress circuits via the vagus nerve and systemic circulation, completing a feedforward loop in which stress alters the microbiome and gastrointestinal symptoms amplify psychological distress (Steinert et al., 2017).

Peripheral CRH also sensitizes visceral nociceptors, lowering the threshold for abdominal discomfort and pain perception. Mast cell degranulation in the colonic mucosa releases histamine, tryptase, and prostaglandins that activate silent nociceptors and increase afferent fiber firing rates (Taché and Bonaz, 2007). This sensitization may explain why gastrointestinal symptoms in astronauts often exceed what objective measures of motility disturbance would predict. The dissociation between subjective symptom severity and objective physiological impairment highlights the critical role of central processing in spaceflight gastrointestinal disorders. Targeted interventions that modulate vagal tone or normalize HPA axis responsiveness may prove more effective than purely peripheral approaches for managing brain-gut axis dysfunction during long-duration missions.

## 6 Humoral factors modulating gastrointestinal motility under microgravity

### 6.1 Impaired prokinetic hormone secretion: temporal dynamics

Gastrointestinal hormones serve as critical humoral regulators of gut motility. Under microgravity, multiple prokinetic hormones exhibit reduced secretion or attenuated function. Ghrelin, produced predominantly by the gastric fundus, stimulates appetite and accelerates gastric emptying (Steinert et al., 2017). In simulated microgravity, rats demonstrate decreased plasma ghrelin levels, attributable to altered gastric fundus distension and aberrant vagal modulation (Chen et al., 2012). Chen and colleagues reported that tail-suspension rats exhibited reduced plasma ghrelin under simulated weightlessness, with partial recovery observed at 21 days compared with 14 days (Chen et al., 2012). This reversible pattern implies an adaptive self-regulatory response.

Motilin initiates the interdigestive migrating motor complex (MMC) phase III. Li and colleagues observed that serum gastrin concentrations in tail-suspended rats increased significantly at 6 and 12 hours, then gradually declined to near-baseline levels by day 5, whereas motilin levels rose continuously throughout the suspension period (Li et al., 2013). This divergent temporal response suggests fundamentally distinct adaptive mechanisms. The early gastrin surge likely reflects acute stress activation; persistent motilin elevation may relate to MMC rhythm disruption and impaired bacterial clearance.

Serotonin (5-HT), synthesized by enterochromaffin cells, is essential for enteric nervous system (ENS) excitation and interstitial cell of Cajal (ICC) pacemaker regulation. The cited literature establishes this general physiology (Gershon and Tack, 2007; Yano et al., 2015) but does not demonstrate that microgravity reduces intestinal 5-HT synthesis; the direction and timing of 5-HT changes in flight remain an evidence gap.

### 6.2 Anti-motility hormones: model-dependent alterations

At the same time, microgravity may alter secretion of anti-motility hormones. Glucagon-like peptide-1 (GLP-1), released by L-cells in the distal ileum and colon, potently inhibits gastric emptying (Steinert et al., 2017). Whether GLP-1 secretion itself changes during spaceflight has not been directly measured; its relevance here rests on this established inhibitory physiology and on the reduced food intake consistently reported in astronauts (Riepl et al., 2002; Steinert et al., 2017). Cholecystokinin (CCK), released during fat digestion, suppresses gastric emptying and increases pyloric tone (Steinert et al., 2017).

Vasoactive intestinal peptide (VIP) and secretin are upregulated in simulated microgravity, both inhibiting gastrointestinal smooth muscle contraction. Pancreatic polypeptide (PP), secreted in response to vagal stimulation, suppresses gut motility and secretion (Riepl et al., 2002; Steinert et al., 2017). Somatostatin functions as a master regulator of gastrointestinal hormone activity; direct measurements under microgravity are scarce and inconsistent. Zhu and colleagues reported that somatostatin-immunoreactive cells in the gastric antrum decreased significantly after 14 and 28 days of simulated weightlessness, alongside reduced interleukin-2 immunoreactivity (Zhu et al., 2004). This finding appears paradoxical. Reduced local somatostatin expression may, however, disinhibit gastrin secretion, indirectly contributing to gastric acid hypersecretion rather than directly enhancing motility. Wang and colleagues further demonstrated increased gastric mucosal leptin and leptin receptor expression after 28 days of simulated weightlessness (Wang et al., 2007).

Taken together, the evidence does not support one stable hormonal signature of microgravity, such as uniform prokinetic insufficiency with inhibitory excess; hormonal dysregulation is a plausible component of altered motility, but its direction is model-, compartment- and time-dependent. Riepl and colleagues monitored astronauts during the EUROMIR-94 mission: acute microgravity exposure increased VIP, PP, motilin, and secretin while decreasing CCK; after four weeks of chronic exposure, CCK, motilin, VIP, neurotensin, and insulin increased, whereas gastrin, PP, secretin, and somatostatin showed no significant change (Riepl et al., 2002). Several inconsistencies in the literature warrant caution. Different studies report divergent directional changes for the same hormones, with some exhibiting an acute elevation followed by chronic recovery. Most observations derive from plasma concentrations, which may not reflect local tissue levels or receptor sensitivity at the enteric neural plexus. The limited efficacy of exogenous prokinetic agents in ground-based simulations further suggests that structural cellular damage—ICC apoptosis, tight junction disruption—may represent a more fundamental limiting factor than hormonal imbalance alone.

## 7 Cellular and molecular mechanisms of microgravity-induced motility impairment

### 7.1 Interstitial cells of cajal: pacemaker network disruption

ICC generate slow waves and coordinate smooth muscle contraction rhythms. Through gap junctions, ICC form a multicellular syncytium with smooth muscle cells and PDGFRα+ cells, maintaining close communication with enteric neurons. ICC differentiation, maintenance, and function depend upon the stem cell factor (SCF)/c-kit signaling axis; disruption of this pathway produces gastrointestinal dysmotility (Chen et al., 2008a; Li et al., 2011).

Evidence from simulated models indicates that microgravity damages ICC via oxidative stress pathways. In these models, space radiation and microgravity-induced reactive oxygen species (ROS) accumulation disrupt ICC mitochondrial function, leading to downregulated c-kit expression and loss of pacemaker activity (Li, 2008). ICC exhibit particular vulnerability to oxidative stress; depletion of heme oxygenase-1 (HO-1) antioxidant defense triggers ICC apoptosis and network fragmentation (Li, 2008; Casero et al., 2017). Li and colleagues reported decreased c-kit protein and mRNA levels in the gastric antrum of simulated weightless rats, implicating impaired ICC pacemaker function as a proximal cause of gastric dysmotility (Li et al., 2013). Whether the same sequence of ICC injury occurs in human orbital flight has not been demonstrated.

The reversibility of ICC damage remains unresolved. Network repair requires substantial time even after return to normal gravity, which may explain why some astronauts experience persistent gastrointestinal symptoms post-landing (Li, 2008). A recent study demonstrated that *Bifidobacterium animalis* subsp. *lactis* BLa80 improved gut microbiota and mucosal status in simulated microgravity rats via SCF/c-Kit signaling modulation (Zhang et al., 2025). This finding raises the possibility that probiotic intervention may exert direct effects on ICC molecular repair, extending beyond the established microbiota-metabolite axis.

#### 7.1.2 Candidate oxidative and mitochondrial pathways

The molecular basis of ICC vulnerability to microgravity involves dysregulated redox homeostasis. Under normal conditions, the Nrf2/ARE pathway maintains antioxidant defense by upregulating SOD, catalase, and GPx. Microgravity suppresses Nrf2 nuclear translocation and reduces ARE-driven transcription of these antioxidant enzymes (Li, 2008). Concurrently, mitochondrial dysfunction—characterized by impaired respiratory complex I and III activity, reduced mitochondrial membrane potential, and increased electron leak—generates excessive superoxide anions that overwhelm residual scavenging capacity. The resulting oxidative shift damages mitochondrial DNA and disrupts ATP-dependent ICC pacemaker currents. These endpoints are extrapolated from the broader space-biology literature and have not been measured in gastrointestinal ICC under altered gravity; they should be read as testable mechanisms rather than established events.

Autophagy, the primary cellular mechanism for clearing damaged mitochondria (mitophagy), is similarly compromised under microgravity. Reduced expression of autophagy-related proteins (LC3-II, Beclin-1, Atg5) and impaired lysosomal degradation efficiency lead to accumulation of dysfunctional mitochondria, further exacerbating ROS production (Li, 2008). The dual impairment of antioxidant defense and autophagic clearance creates a cellular environment in which ICC cannot maintain structural integrity.

### 7.2 Smooth muscle dysfunction and intestinal barrier disruption

Smooth muscle cells are the final effector cells of gastrointestinal motility. Under microgravity, oxidative stress and inflammatory cytokines (TNF-α, IL-6) impair L-type calcium channel function on the smooth muscle membrane and disrupt Ca²^+^ release from the sarcoplasmic reticulum, resulting in reduced Ca²^+^ transient amplitude and rhythmic disturbance. Prolonged hypokinetic states cause smooth muscle atrophic remodeling, with reduced intestinal and colonic smooth muscle layer thickness and downregulated expression of contractile proteins (Li et al., 2008).

Intestinal barrier integrity is essential for normal gastrointestinal motility. Under microgravity, epithelial tight junction proteins (occludin, claudins, ZO-1, E-cadherin) are downregulated (Turner, 2009; Jin et al., 2018), while the TLR4/MyD88/NF-κB signaling axis is activated, promoting pro-inflammatory cytokine transcription (Li et al., 2008). Myosin light chain kinase (MLCK) is upregulated, driving myosin light chain phosphorylation, disrupting the actin cytoskeleton, and causing tight junction protein translocation from the cell membrane to the cytoplasm (Li et al., 2008).

Different studies reveal dynamic temporal patterns in tight junction disruption that carry methodological significance. Li and colleagues found that ZO-1 and occludin expression decreased significantly in tail-suspended rats (Li et al., 2021). Alvarez and colleagues, using a clinostat to simulate microgravity in cultured intestinal epithelial cells, observed that ZO-1 and occludin decreased significantly at day 9 but recovered to control levels by day 11, suggesting that microgravity may delay protein trafficking and junction assembly rather than reduce total protein expression (Alvarez et al., 2019). Chen and colleagues further demonstrated through immunohistochemistry and RT-qPCR that occludin and ZO-1 showed continuous but uneven distribution along villi at 14 days, with significantly reduced density compared with controls; by 21 days, further reduction occurred with discontinuous, patchy distribution, and significantly decreased mRNA expression (Chen et al., 2011).

Integrating these observations, microgravity appears to produce a progressive sequence: acute junction assembly delay followed by chronic expression downregulation. Short-term clinostat exposure (9–11 days) likely reflects cytoskeletal remodeling and protein trafficking impairment, whereas long-term tail-suspension models (14–21 days) involve transcriptional suppression and disturbed epithelial turnover. This temporal distinction suggests short-duration spaceflight may primarily cause reversible barrier functional impairment, whereas long-term habitation risks structural barrier defects.

Even partial-gravity environments may not fully normalize epithelial barrier function. A 2026 *in vitro* study found that simulated Martian gravity (0.38 g) altered tight-junction components and impaired epithelial barrier integrity (Benvenuti et al., 2026). These findings raise the hypothesis that partial gravity may be insufficient for complete barrier recovery; whether this translates into persistent barrier or motility abnormalities after planetary landing requires ***in vivo*** and human validation.

### 7.3 Small intestinal mucosal morphological changes and apoptosis

Microgravity directly damages small intestinal mucosal architecture. Chen and colleagues demonstrated that after 14 and 21 days of tail suspension, rats exhibited reduced villus number, significantly decreased villus height, and shallower crypts, with villus twisting, adhesion, and fusion evident under light microscopy. Electron microscopy showed reduced microvillus height, increased width, reduced surface area, and thinner glycocalyx (Chen et al., 2008b). Shortened villi and reduced microvillus surface area directly diminish the absorptive surface. Impaired absorption alters luminal physicochemical properties, indirectly disturbing gastrointestinal motility rhythms.

Small intestinal mucosal structure and barrier function damage fundamentally stem from epithelial apoptosis. Li and colleagues showed that simulated weightlessness induced significant small intestinal epithelial cell apoptosis after merely 1 day, with elevated apoptosis persisting above control levels at 7 days (Li, 2008). NF-κB, a central mediator of immune-inflammatory responses, began rising at 0.5 days, peaked at 2 days, and remained significantly elevated at 21 days, driving production of IL-6, IL-8, and TNF-α (Li et al., 2008). This early initiation of the apoptosis-inflammation cascade indicates that intestinal damage occurs almost immediately upon microgravity exposure rather than accumulating gradually. The temporal profile supports early prophylactic intervention upon entry into the space environment.

The cited altered-gravity studies do not directly demonstrate increased mTOR phosphorylation, reduced AMPK activity or impaired autophagic flux in intestinal epithelium; these candidate pathways should be evaluated prospectively rather than described as established components of the syndrome. If confirmed, the interplay between autophagy suppression and apoptosis acceleration would constitute a dual-hit mechanism compounding mucosal barrier disruption (Li, 2008).

### 7.4 Impaired immune barrier function

Microgravity induces systemic immunosuppression that compromises intestinal mucosal immune barrier function. Li and colleagues demonstrated that after 1 day of simulated weightlessness, rats showed significant lymphocyte apoptosis in the mucosa, reduced CD4^+^ T cells, and decreased secretory IgA (sIgA) levels in the small intestine. By 7 days, sIgA secretion partially recovered, lymphocyte apoptosis declined, and CD4^+^ T cell numbers increased, though all remained significantly below control levels (Li, 2008). This “acute collapse—partial recovery” pattern indicates that the intestinal immune barrier possesses limited compensatory capacity.

The mechanism likely involves TLR/NF-κB signaling activation. Simulated weightlessness may alter gut microbiota structure, increasing pathogen-associated molecular pattern (PAMP) expression and activating the TLR4/MyD88/NF-κB cascade, thereby inducing mucosal immune dysregulation (Li et al., 2008). These interdependencies create a self-perpetuating cycle of barrier damage, bacterial translocation and inflammation that further injures the barrier and ICC networks (Section 8.1). Interrupting this cycle at multiple points—through probiotic administration, antioxidant supplementation, and tight junction stabilization—represents a rational multimodal strategy for long-duration spaceflight.

## 8 Gut microbial mechanisms underlying gravitational effects on gastrointestinal motility

### 8.1 Dysbiosis: from consequence to causative factor

The intestinal microbiota constitutes a critical determinant of gastrointestinal motility homeostasis. Microgravity perturbs this equilibrium through several converging pathways. Delayed gastric emptying and disrupted migrating motor complex (MMC) rhythms promote small intestinal bacterial overgrowth (SIBO) (Deloose et al., 2012). Alterations in mucus composition and epithelial barrier integrity further compromise the niche required by commensal populations (Chen et al., 2008b).

During low Earth orbit missions, astronauts are exposed to solar particle events and galactic cosmic radiation. Radiation and microgravity are concurrent mission stressors whose gastrointestinal effects should be evaluated separately as well as in combination. Butyrate-producing taxa—including *Faecalibacterium prausnitzii*, *Roseburia*, and *Blautia*—demonstrate particular sensitivity to oxidative stress, exhibiting markedly reduced abundance under spaceflight conditions (Turroni et al., 2020). A 2024 investigation confirmed that radiation combined with simulated microgravity exerts joint tumour-promoting effects in intestinal tissue (Suzuki et al., 2024); that endpoint was tumorigenesis rather than the microbiome, so synergistic microbiome injury remains to be demonstrated. Even so, ground-based studies relying solely on microgravity simulation may underestimate the severity of dysbiosis encountered during actual spaceflight.

Voorhies et al. followed nine astronauts during long-duration International Space Station (ISS) missions, with in-flight *fecal* samples available for five (Voorhies et al., 2019). Gastrointestinal alpha diversity and richness increased during flight in four of the five stool-sampled astronauts, while community composition shifted and several potentially anti-inflammatory genera, including *Akkermansia* and *Ruminococcus*, declined. Most changes moved towards baseline after return (Voorhies et al., 2019). Shared diet and environment, small sample size and *fecal* sampling limit causal interpretation. The reversibility of these shifts suggests that dysbiosis under microgravity represents primarily an environmentally driven adaptive response rather than a genetic alteration in host-microbe interactions.

This reversibility should not, however, be interpreted as harmless. Whether microgravity enhances microbial resistance to antimicrobial agents remains unresolved. Low-shear and spaceflight experiments can alter microbial stress responses, virulence or antibiotic susceptibility in some organisms (Taylor, 2015), but the available evidence does not establish a consistent increase in minimum inhibitory concentrations among resident gut isolates from astronauts. Infection-related implications should therefore be described in general, organism-specific terms.

The relationship between dysbiosis and dysmotility is bidirectional and temporally coupled. Gastric emptying delay and MMC disruption cause small bowel stasis, creating favorable conditions for bacterial overgrowth. Conversely, altered microbial metabolites feed back onto interstitial cells of Cajal (ICC) and enteric nervous system (ENS) function, compounding motility deficits. Murine hindlimb unloading models demonstrate that gastrointestinal transit slowing and compositional shifts in gut microbiota emerge within three days of exposure, indicating close temporal coupling (Shi et al., 2017). In this causal chain, motility disturbance likely functions as the initiating event, with dysbiosis serving as an amplifying and sustaining factor—a self-reinforcing loop that perpetuates gastrointestinal dysfunction in the absence of gravitational loading. Recent human data support this coupling: longitudinal plasma metabolomics from 52 ISS astronauts revealed increased circulating markers of gut microbial protein fermentation during flight; the authors considered this pattern possibly consistent with prolonged intestinal transit, although transit was not directly measured (La Barbera et al., 2026).

The reliance on fecal microbiota as a proxy for the entire gastrointestinal microbial landscape remains a significant limitation. Fecal samples predominantly reflect colonic rather than small intestinal communities, yet the proximal gut may be the primary site of microgravity-induced dysbiosis. Transcriptomic and metabolomic profiling of mucosal-associated microbiota, which remain technically challenging during flight, would provide more mechanistic insight.

### 8.2 Probiotic and prebiotic interventions: from ground evidence to space application

The reversibility of microgravity-induced dysbiosis suggests that targeted microbial interventions may offer viable countermeasures. Among the most promising candidates, *Bifidobacterium lactis BLa80* has demonstrated protective effects against simulated microgravity-induced ICC damage through the stem cell factor (SCF)/c-kit signaling pathway, partially restoring pacemaker function in cultured ICCs (Zhang et al., 2025). This mechanism directly links microbial supplementation to the restoration of the very cells governing gastrointestinal rhythmicity.

*Hericium erinaceus* polysaccharides have shown gastroprotective properties in tail-suspension rat models, ameliorating gastrointestinal dysfunction through modulation of mucosal barrier integrity and inflammatory responses (Zang et al., 2025).

The translation of probiotic interventions from terrestrial to space environments faces three specific obstacles. First, microbial viability during long-duration storage and fluctuating radiation exposure on spacecraft demands formulation optimization, including microencapsulation and lyoprotectant strategies. Second, the individual variability in baseline microbiota composition among astronauts suggests that personalized probiotic or synbiotic regimens, tailored to pre-flight microbial profiling, may prove more effective than universal formulations. Third, the limited number of flight-validated studies restricts evidence-based prescribing. Current ISS investigations of probiotic supplementation have been short-term and underpowered for clinical endpoints; definitive trials with motility-related primary outcomes are needed.

Prebiotic substrates warrant parallel consideration. Dietary fibers that selectively enrich butyrate-producing taxa—such as resistant starch and galacto-oligosaccharides (GOS)—may indirectly support ICC function through SCFA-mediated mechanisms. Direct evidence from altered-gravity models, though limited to a single study, is encouraging: in female mice exposed to 3 g centrifugation, a prebiotic diet containing GOS and polydextrose increased Bifidobacterium abundance and prevented the hypergravity-induced shifts in β-diversity and the expansion of Clostridium sensu stricto 1 and Turicibacter, while also reducing the neutrophil-to-lymphocyte ratio (Thompson et al., 2025). Whether GOS-based regimens afford similar protection under simulated or actual weightlessness has not yet been tested. The efficacy of such prebiotic approaches under conditions where endogenous butyrate producers are already depleted by radiation exposure remains uncertain, however, and represents a priority for pre-flight validation. It should be emphasized that all interventions discussed in this section remain investigational. The supporting evidence derives almost entirely from terrestrial disease models and ground-based simulations, and none has been validated against motility endpoints during actual spaceflight.

### 8.3 SCFA and bile acid metabolic disturbances

Short-chain fatty acids (SCFAs), particularly butyrate, serve as the principal energy substrate for colonic epithelial cells and are essential for maintaining mucosal barrier integrity and colonic contractility. Microgravity-induced reduction in butyrate-producing taxa directly diminishes SCFA output (Turroni et al., 2020). Clinical studies in slow-transit constipation demonstrate significantly lower fecal butyrate and propionate concentrations relative to healthy controls, establishing a disease-relevant benchmark for astronaut dysbiosis.

SCFAs promote colonic motility through several distinct mechanisms: direct activation of enteric neurons via FFAR2/FFAR3 receptors; enhanced 5-HT synthesis by enterochromaffin cells; strengthened ICC pacemaker function through SCF/c-Kit signaling; and inhibition of inhibitory neurotransmitter release (VIP and nitric oxide) within the ENS (Turroni et al., 2020; Li et al., 2021). SCFA deficiency therefore produces convergent impairment of smooth muscle energetics, neuronal excitability, and ICC pacemaking capacity.

Beyond SCFAs, bile acid metabolism represents a second microbial pathway disrupted by microgravity. Simulated unloading alters not only microbial composition but also the spectrum of bile acid metabolites generated by bacterial 7α-dehydroxylation and epimerization (Wang et al., 2024). Bile acids function as signaling molecules through TGR5 and FXR receptors, modulating intestinal peristalsis and barrier function. Dysbiosis-driven shifts in the bile acid metabolome may therefore further perturb colonic contractile rhythms and absorptive-secretory balance, adding a layer of metabolic dysregulation to the motility deficit.

In the astronaut study by Voorhies et al., changes in *Fusicatenibacter* and *Dorea* abundance were associated with selected circulating cytokines (Voorhies et al., 2019). These associations link microbial composition and host inflammation, but they do not demonstrate reduced SCFA output or a causal pathway to dysmotility.

The assumption that fecal SCFA concentrations reliably reflect luminal availability in the proximal colon and small intestine may not hold under microgravity, where regional transit times are differentially affected. Direct luminal sampling or stable-isotope tracer studies would clarify this relationship.

### 8.4 Microbiota-gut-brain axis and gut-liver axis perturbations

The intestinal microbiota exerts modulatory influence over ENS and central nervous system function through neurotransmitter production (5-HT, GABA, dopamine) and metabolite generation (SCFAs, tryptophan derivatives) (Yano et al., 2015; Li et al., 2021). Microgravity-induced dysbiosis alters the availability of these signaling molecules, which reach central autonomic centers via vagal afferents and immune-mediated pathways. Reduced butyrate-producer abundance diminishes tryptophan metabolites—indole derivatives that serve as aryl hydrocarbon receptor (AhR) ligands critical for intestinal immune homeostasis and ENS integrity (Yano et al., 2015). The resulting loss of AhR-mediated signaling may compromise enteric glial cell function and neuro-epithelial communication.

Recent evidence from long-term tail-suspension models indicates that gut-liver axis homeostasis is disrupted in simulated weightlessness, with abnormal hepatic metabolic enzyme expression and lipid metabolism disturbance (Chen et al., 2026). This expands weightlessness-induced motility disorder research from “intestinal local” to “gut-liver integrated” perspectives. Intestinal barrier disruption enables translocation of bacterial endotoxins and metabolites to the hepatic portal circulation, activating Kupffer cells and triggering hepatic inflammatory responses. Concurrently, altered bile acid secretion from the liver reshapes the luminal environment for microbial colonization, potentially selecting for pro-inflammatory taxa and further impairing intestinal motility. This creates a triangular circuit—gut dysbiosis, hepatic metabolic disturbance, and altered enterohepatic bile acid signaling—each vertex of which can independently and synergistically affect motility.

A 2025 study further demonstrated that microgravity-induced intestinal flora dysbiosis propagates to liver and brain metabolism through specific molecular signaling pathways (Xiong et al., 2025). Whether these effects are mediated primarily by circulating microbial metabolites, extracellular vesicles, or immune cell activation remains unresolved.

Existing studies of the microbiota-gut-brain and gut-liver axes in microgravity remain largely correlational. Causal inference is hampered by the practical impossibility of performing fecal microbiota transplantation or germ-free animal experiments in orbital laboratories. Ground-based rotating wall vessel and random positioning machine models offer partial alternatives but cannot replicate the full spectrum of space stressors. Establishing causal directionality—whether dysbiosis drives motility disturbance or vice versa—represents the most pressing mechanistic question in this domain.

## 9 Conclusions and future directions

### 9.1 Integrative summary of mechanistic pathways

Gravitational unloading disrupts gastrointestinal motility through five overlapping mechanisms: altered intra-abdominal mechanical loading, disturbed autonomic and enteric neural regulation, reorganized gastrointestinal hormone secretion profiles, oxidative stress-induced damage to ICC and smooth muscle, and dysbiosis with attendant metabolite derangement. A firm temporal ordering has not been established: microbial changes can appear within the first flight week or within days in animal models (Shi et al., 2017; Voorhies et al., 2019), whereas the onset and reversibility of ICC changes are poorly characterized. Whether fibrotic or epigenetic alterations accumulate over months remains unknown—no study has tracked histological changes beyond 28 days of simulated unloading. This temporal heterogeneity implies that the dominant driver of dysmotility shifts with mission duration, a feature that single-timepoint studies invariably miss.

The current evidence base suffers from a fundamental disconnection between mechanistic sophistication and ecological validity: ground-based models yield detailed molecular insights but capture only selected aspects of spaceflight, whereas flight-based investigations remain constrained by small samples and indirect endpoints (see Section 9.3).

### 9.2 Knowledge gaps and specific research hypotheses

Several critical questions remain unanswered. First, the temporal primacy of ICC damage relative to neural dysfunction and hormonal dysregulation has not been established. The hypothesis that microgravity-induced oxidative stress in ICC mitochondria constitutes the initiating event—subsequently triggering secondary neural adaptation and hormonal compensatory responses—warrants direct testing using time-resolved single-cell transcriptomics in flight-validated models.

Second, the quantitative contribution of radiation to gastrointestinal dysmotility, independent of and synergistic with microgravity, is unknown. Ground-based studies employing combined radiation and unloading exposure would clarify whether current countermeasures targeting microgravity alone are fundamentally insufficient for deep-space missions.

Third, the reversibility of ICC structural damage following prolonged exposure remains controversial. Studies should first distinguish apoptosis, phenotypic loss of c-kit expression and reversible network *remodeling*; only then can a mission-duration threshold, or the need for pharmacological and cell-based interventions, be meaningfully defined for Mars-class mission planning.

Fourth, the causal directionality between dysbiosis and dysmotility in microgravity has not been resolved. Fecal microbiota transplantation from spaceflight-exposed donors into germ-free recipients, with subsequent motility phenotyping, would provide decisive evidence.

Fifth, the effects of hypergravity—an obligatory component of launch, re-entry and parabolic flight—on gastrointestinal motility are essentially unstudied (Section 2.3). Within-subject comparison across the gravity spectrum would clarify whether gravitational loading and unloading produce symmetric or qualitatively distinct motor phenotypes.

### 9.3 Limitations of current evidence

The field faces three methodological limitations that constrain the confidence with which findings can be extrapolated to human spaceflight. First, model validity is incomplete: head-down tilt bed rest reproduces fluid shifts but not three-dimensional organ flotation or cosmic radiation; hindlimb unloading imposes non-physiological restraint stress; and rotating wall vessel cultures lack systemic neurohumoral inputs. No single model captures the integrated pathophysiology of spaceflight.

Second, mechanistic research has proceeded in fragmented fashion. Most investigations examine isolated pathways—a single hormone, a specific protein, or an individual microbial genus—yet gravitational dysmotility arises from network-level perturbation in which ICC damage, barrier dysfunction, dysbiosis, and inflammatory signaling are dynamically interdependent. The relative weighting of these nodes and their causal ordering remain undefined.

Third, intervention development lags behind mechanism discovery. Existing countermeasures—physical exercise protocols, generic probiotic supplementation, and prokinetic pharmacotherapy—were designed for terrestrial indications and have not been validated against space-specific endpoints. The irreversibility of ICC structural damage and the radiation-microgravity synergy are rarely incorporated into preclinical countermeasure testing.

### 9.4 Priorities for future research

Future investigation should prioritize four objectives. First, establish multi-factorial simulation platforms that integrate radiation exposure, vestibular stimulation, psychological stress, and altered gravitational loading, alongside strengthened longitudinal cohort studies during actual long-duration flights. Second, elucidate the temporal sequence of ICC molecular damage under combined radiation and unloading conditions, with particular attention to SCF/c-Kit, 5-HT, and redox signaling pathway interactions, and develop non-invasive ICC functional assessment methods suitable for human validation. Third, characterize the role of the gut-liver axis in motility regulation, integrating hepatobiliary function with intestinal barrier and microbial assessments. Fourth, advance precision microbiome interventions—including strain-specific probiotics such as BLa80 targeting ICC repair (Zhang et al., 2025), natural products with gastroprotective properties (Zang et al., 2025), and individualized prebiotic formulations informed by astronaut microbial profiling, all of which remain investigational and are supported mainly by preclinical evidence—while evaluating barrier integrity under partial gravity (e.g., Martian 0.38 g) as a determinant of countermeasure efficacy for deep-space missions.

The development of an integrated countermeasure framework—encompassing physical protection, cellular preservation, and microbiome modulation—will be essential for safeguarding crew health as human exploration extends beyond low Earth orbit.
