# Supplementary material for: Gastrointestinal motility in microgravity: a critical review of multi-level mechanisms and model-dependent effects
Source: Front Physiol. 2026 Aug 20;17:1930628. doi: 10.3389/fphys.2026.1930628 (PMC13539599; doi:10.3389/fphys.2026.1930628)
Supplement: Supplementary file 1 [file Supplementaryfile1.docx]

**Supplementary Material**

*Manuscript: Gastrointestinal Motility in Microgravity: A Critical Review of Multi-Level Mechanisms and Model-Dependent Effects*

**Supplementary Figure S1.** Anatomical reference map of the principal motility regulators discussed in this review. The stomach, small intestine and colon are shown with representative pacemaker cells, neurotransmitters, hormones and microbial signals cited in the manuscript. Green labels denote predominantly prokinetic or excitatory factors; red labels denote predominantly inhibitory factors; black labels denote structural or functional components. Arrows indicate physiological regulatory relationships and should not be interpreted as uniform directional changes during microgravity. The possible MMC-disruption/SIBO relationship and the dysbiosis-stasis feedback loop are model-dependent or hypothesis-generating. Extrinsic neural and stress-related inputs act from above, and luminal microbial signals act from below.

Abbreviations: 5-HT, 5-hydroxytryptamine; ACh, acetylcholine; CCK, cholecystokinin; GAS, gastrin; GLP-1, glucagon-like peptide-1; HAPC, high-amplitude propagating contractions; HPA, hypothalamic-pituitary-adrenal; ICC, interstitial cells of Cajal; MMC, migrating motor complex; MTL, motilin; NO, nitric oxide; SCFA, short-chain fatty acids; SP, substance P; VIP, vasoactive intestinal peptide.

The schematic is original to this review and is intended as an orientation aid for the reader, not as a quantitative summary.
